# Identification of a Mutation in FGF23 Involved in Mandibular Prognathism

**DOI:** 10.1038/srep11250

**Published:** 2015-06-10

**Authors:** Fengshan Chen, Qin Li, Mingliang Gu, Xin Li, Jun Yu, Yong-Biao Zhang

**Affiliations:** 1 Laboratory of Oral Biomedical Science and Translational Medicine, School and Hospital of Stomatology, Tongji University, Shanghai, P. R. China; 2 Beijing Institute of Genomics, Chinese Academy of Sciences and Key Laboratory of GenomeScience and Information, Chinese Academy of Sciences, Beijing, P. R. China; 3 Department of Cardiology, Beijing Anzhen Hospital of the Capital University of Medical Sciences, Beijing, P. R. China

## Abstract

Mandibular prognathism (MP) is a severe maxillofacial disorder with undetermined genetic background. We collected a Chinese pedigree with MP which involved in 23 living members of 4 generations. Genome-wide linkage analysis were carried out to obtain the information in this family and a new MP-susceptibility locus, 12pter-p12.3 was identified. Whole-exome sequencing identified a novel heterozygous mutation in *fibroblast growth factor (FGF)* 23 (; p.A12D) which well segregated with MP in this pedigree within the locus. The mutation was also detected in 3 cases out of 65 sporadic MP patients, but not in any of the 342 control subjects. The p.A12D mutation may disrupt signal peptide function and inhibit secretory in FGF23. Furthermore, mutant FGF23 was overexpressed in 293T cells, increased cytoplasmic accumulation was observed compared with the wild type. We have discovered that c.35C>A mutation in *FGF23* strongly associated with MP, which expand our understanding of the genetic contribution to MP pathogenesis.

Mandibular prognathism (MP; MIM 176700) is a dentofacial deformity characterized by overgrowth of the lower jaw with or without undergrowth of the upper jaw^1^. The discrepancy between the upper and lower jaw can cause a deficiency in speech articulation and low masticatory efficiency^2^. Epidemiological data indicate that MP prevalence rates range from 0.48% to 4.3% in Caucasian populations and from 2.1% to 10% in Chinese populations^3^,^4^,^5^. According to OMIM, MP can occur as non-syndromic condition or as one phenotype of systemic diseases, such as Apert syndrome and Crouzon syndrome.

Numerous risk factors have been reported in association with MP. Both genetic and environmental factors contribute to this occlusion disorder^1^,^5^. To date, 11 common genetic loci have been reported to be associated with MP^6^,^7^,^8^,^9^,^10^, including 1p22.1, 1q32.2, 3q26.2, 11q22, 12q13.13, 12q23, 1p36, 6q25, 19p13.2, 14q24.3-31.2, and 4p16.1. Moreover, 1p22.3 and 1q32.2 have also been reported to be associated with MP using genome-wide association study (GWAS)^6^. Among these studies, Yamaguchi *et al* and Li *et al* investigated largely on the mandibular prognathic subtype^8^,^9^,^10^, whereas Frazier-Bowers *et al* found that affected individuals were mostly maxillary deficient^7^. There also have a host of genes which might influence MP, such as: *GHR*^11^, *EPB41*^12^, *MATN1*^13^, *MYO1H*^14^. Recently, Nikopensius *et al*^15^ performed whole-exome sequencing on five siblings from an Estonian family affected by class III malocclusion and identified a mutation of *DUSP6*, c.545C>T (p.Ser182Phe), which is likely a causal variant of class III malocclusion. The family members they studied exhibited maxillary retrusion or mandibular protrusion. Due to the highly variable subphenotypes (mandibular protrusion, maxillary retrusion, or a combination of both) and clinical heterogeneity of MP, genetic mechanism for this malformation remains uncertain.

In this study, we focus on simple mandibular prognathism without maxillary retrognathism. Genome-wide linkage scans and exome sequencing were carried out to obtain the information in this MP family to provide characteristic etiology for the further delineation of MP.

## Results

### Linkage analysis of a severe MP pedigree

**Figure 1:**
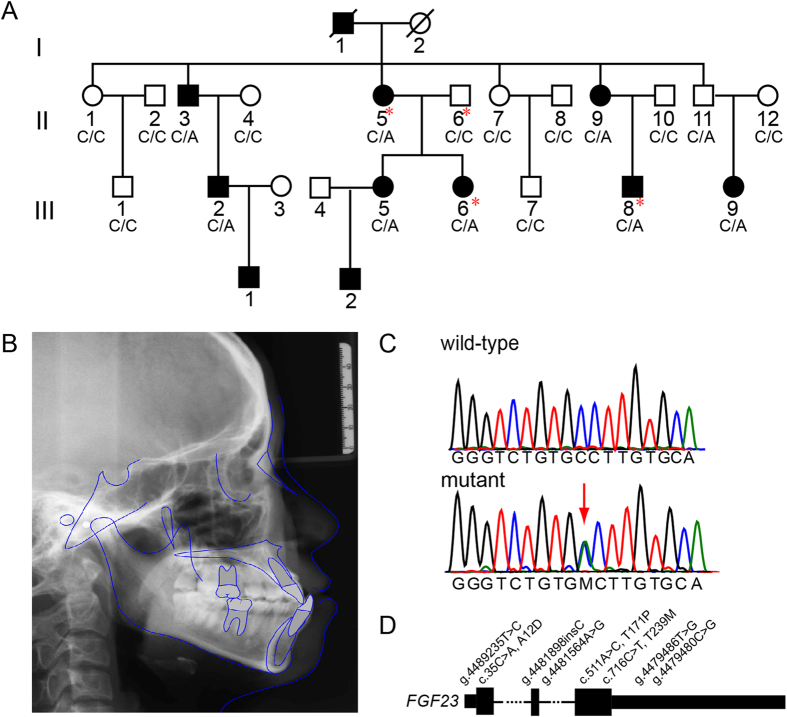
The mandibular prognathism pedigree and its associated genetic variant,c.35C>A. (**A**) MP pedigree evaluated in this study. The genotype with respect to *FGF23* c.35C>A is shown under 19 collected individuals. Four individuals (red asterisks)were chosen for whole-exome sequencing. (**B**) A representative lateral cephalometric tracing from MP patient II1. (**C**) Validation of the c.35C>A mutation (red arrow) using Sanger sequencing. (**D**) All identified *FGF23* mutations from the MP patients in this study (genome reference, hg19).

**Figure 2:**
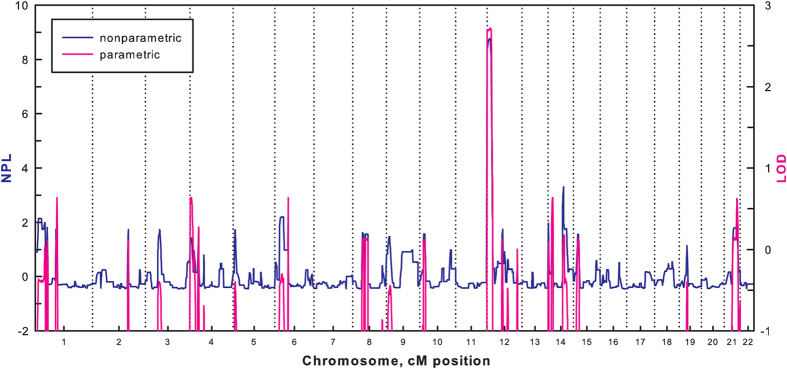
Genome-wide parametric and nonparametric linkage results of the MP pedigree. In the parametric linkage analysis, an autosomal dominant model with a risk allelepenetrance of 0.90 and a phenocopy rate of 0.05 was assumed.

Blood sample were obtained from 19 individuals of 23 living members of the MP pedigree, including 8 MP patients, 1 carrier (II-11, has a MP daughter)(Table 1), and 10 unaffected members (Fig. 1A). A lateral cephalometric trace of the proband was obtained from MP patient II5 and shown in Fig. 1B. A visual inspection of the pedigree suggested an autosomal dominant mode of disease inheritance. We performed a genome-wide linkage analysis on the MP pedigree using total 4,958 informative autosomal SNPs. Both the parametric and nonparametric analyses indicated the highest linkage scores on chromosome 12pter-p12.3 (NPL = 8.68, LOD = 2.705) (Fig. 2). Both of our highest parametric and nonparametric linkage score, simulated 0.44 and 0.45 times per genome scan, fall within the range of suggestive linkage signal, according to criteria proposed by Lander & Kruglyak^16^. No other suggestive linkage signals were observed in the genome. In brief, our linkage analysis identifies a putative linkage signal for the MP pedigree on chromosome 12pter-p12.3.

### Whole-exome sequencing

Exomes of 3 affected individuals (II5, III6, and III8) and one unaffected one (II6) were sequenced in order to screen the causal genes of the MP pedigree. The exome sequencing had a 42-fold mean coverage and revealed a total of 45,507 single-nucleotide polymorphisms (SNPs), 1158 small insertion/deletions (indels), and 13 structural variations at exome region. We removed variants that had a global minor allele frequency >0.01 in the database of dbSNP138 or 1000 Genomes Project. Considering the dominant-inherited mode of the MP pedigree, total 657 variants were detected in 3 patients but not in the unaffected individual. Furthermore, we focused on variants that result in missense, frameshift, alternative splicing, or within transcription factor-binding sites. Finally, 97 candidate variants (77 SNPs and 20 indels) were screened from 89 genes (Supplementary Table S1). Considering that MP is a bone developmental disease, we narrowed down the gene list to 3 genes of *FGF23*, *FLT3*, and *COL11A2* that maybe involved in skeletal morphogenesis. Consequently, the mutations identified in these genes (*FGF23* c.35C>A, *FLT3* c193A>T and *COL11A2* c.2078G>A) were considered as the most likely causal variants in this MP pedigree.

We further genotyped *FGF23* c.35C>A, *FLT3* c193A>T and *COL11A2* c.2078G>A for all individuals of the MP pedigree. The result showed that only the *FGF23* c.35C>A, located within the susceptibility locus of 12pter-p12.3, well segregated with the MP phenotype (Fig. 1A,C). All 8 patients and the carrier of II11 were heterozygous for this mutation, and other 10 clinically unaffected members did not carry this variant. Therefore, *FGF23* c.35C>A is a potential causal variant in this MP pedigree.

### Screening the *FGF23* gene in the MP pedigree and unrelated MP cases

To detect other *FGF23* variants that may be associated with MP, we sequenced the promoter and coding regions of this gene in the MP pedigree and 65 sporadic MP patients. In total, 8 *FGF23* variants were identified, and 3 of them were predicted to cause amino acid changes (Fig. 1D **and** Supplementary Table S2). Among these variants, c.35C>A was the only one that well segregated with the MP phenotype within the studied pedigree, and it was also detected in 3 of the 65 unrelated cases. All of the 3 sporadic MP patients present high angle of mandibular plane, long body of mandible as the affected ones of the pedigree. To validate that this variant is specific to MP patients, 342 healthy individuals from China were genotyped and none of them were found carried the mutant allele of c.35C>A. In addition, we checked all 8 detected variants in 1000 Genome Project and NHLBI GO Exome Sequencing Projects, and found that c.35C>A was not reported in other world-wide populations (Supplementary Table S3). Therefore, we speculate that the c.35C>A variant is very likely to be the causal mutation of MP in Chinese population.

### Predicted effects of c.35C>A mutation on FGF23 signal peptide

**Figure 3:**
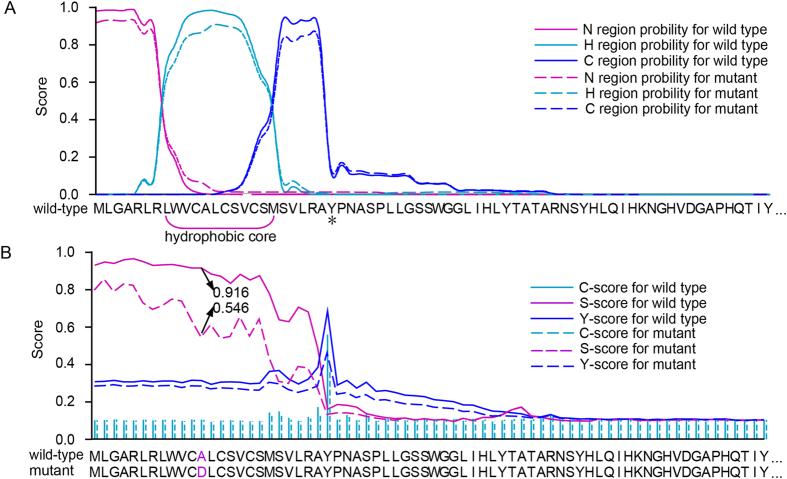
Predict the effects of p.A12D on signal peptide function in the FGF23 protein. (**A**) The 3 functional regions of the signal peptide as determined by SignalP-HMM 2.0. The asterisk marks the first amino acid of the mature FGF23 protein. (**B**)Signal peptide probabilities (measured by the S score) and cleavage-site probabilities(measured by the C and Y scores) of the wild-type and mutant FGF23 proteins were obtained using the SignalP 4.0 software program. The p.A12D mutation reduced the S score of FGF23 from 0.916 to 0.546.

The *FGF23* c.35C>A mutation is predicted to cause a substitution of Asp for Ala in codon 12 (p.A12D) of the FGF23 protein, which is located within the hydrophobic core of the FGF23 signal peptide (Fig. 3A). To evaluate the effects of the p.A12D substitution on signal peptide function, we analyzed the protein sequence of FGF23 using the signal peptide prediction packages SignalP, PrediSi, Signal-CF, and Signal-3L. All packages predicted that the wild-type FGF23 sequence should produce a conventional secretory protein with a cleavage site at the 25Y residue. However, for the mutant FGF23 sequence, Signal-CF and Signal-3L both predicted a shift of the cleavage site, and PrediSi predicted a loss of secretory activity (Supplementary Fig. S1). SignalP predicted that the p.A12D substitution would decrease the C score from 0.56 to 0.37 and Y score from 0.69 to 0.46, which reduce the probability of cleavage site at the 25Y residue. Meanwhile, The S score which measures the signal peptide probabilities was decreased from 0.916 to 0.546 at p.A12D, which results in a decrease of S score at entire signal peptide and harm the capacity of the N-terminus of the nascent FGF23 protein to function as a signal peptide (Fig. 3B). These results suggest that the FGF23 p.A12D substitution may disrupt the translocation of the nascent FGF23 protein to the ER and prevent it from being properly secreted through the ER-Golgi secretory pathway.

### *In vitro* investigation of FGF23 p.A12D substitution

**Figure 4:**
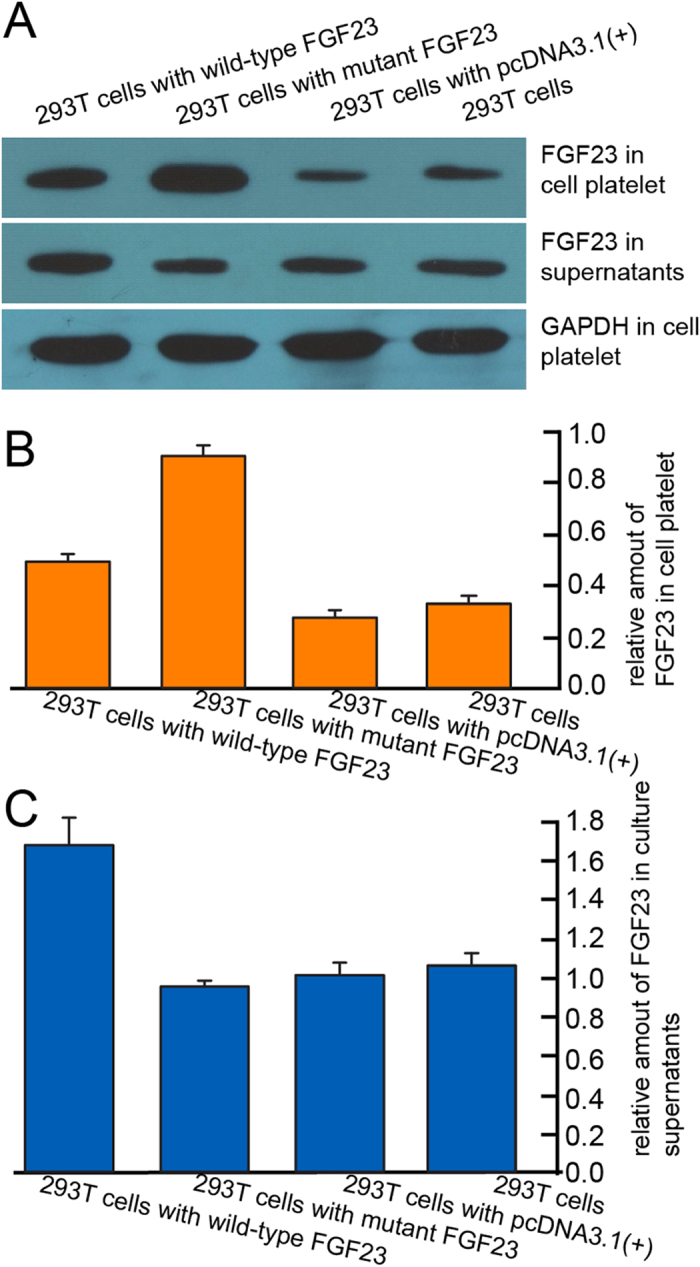
The impact of the p.A12D mutation on the secretion of FGF23. (**A**) Western blotting analysis of the FGF23 immunoprecipitates from the culture supernatants and 293T cells. (**B**) The relative levels of FGF23 in the 293T cells(normalized to GAPDH). (**C**) The relative amounts of FGF23 in the culture supernatants; levels from the cells that were transfected with empty pcDNA3.1(+)vector were set to 1.0.

To investigate the biological impacts of the FGF23 p.A12D mutation, we carried out an *in vitro* assay by overexpressing the wild-type and mutant FGF23 genes in human embryonic kidney 293T cells via transient transfection. The amount of FGF23 in cell lysates and culture medium were detected using immunoprecipitation and subsequent Western blotting (Fig. 4A). The immunoprecipitation of cell lysates from the different cell types revealed approximately 2- to 3-fold higher levels of FGF23 in the cells that were overexpressing the mutant protein compared with those expressing wild-type FGF23 or the negative control cells, respectively (Fig. 4B). The immunoprecipitation of the culture medium showed a 1.6-fold increase in wild-type FGF23 compare with the negative controls (Fig. 4C). In contrast, the intensity of the FGF23 band that immunoprecipitated from the culture supernatant of 293T cells, transfected with the mutant FGF23, was approximately equal to that of the negative controls. These results strongly suggest that the mutant FGF23 protein were not been secreted from 293T cells.

## Discussion

Numerous studies have suggested that there are important genetic factors in the etiology of MP, while few causal mutations had been reported, leaving the genetic basis of this condition unclear. In this study, we performed genome-wide linkage and whole-exome sequencing analyses on an MP pedigree and identified a novel (not reported in dbSNP 138, 1000 Genome Project, or NHLBI GO Exome Sequencing Project) heterozygous mutation in *FGF23* (c.35C>A; p.A12D) that was strongly associated with MP.

Genome-wide linkage studies have reported that many loci were associated with MP^7^,^8^,^17^,^18^, while such claims have been difficult to confirm. Cruz *et al* failed to observe evidence for linkage in previously identified candidate regions in 10 Brazilian families^19^. Tassopoulou-Fishell *et al* studied 8 putative linkage loci in a well-characterized homogeneous sample set and found that only one SNP (rs10850110) within *MYO1H* was associated with MP^20^. In this study, we identified a new genetic locus of 12pter-p12.3 that is associated with MP. The low replication rate for MP-linked loci may result from differences in the genetic backgrounds of the studied populations and the existence of multiple genetic causes of MP^19^. Moreover, the studied MP pedigree is characteristic not only by mandibular prognathism without maxillary retrognathism, but also by high angle of mandibular plane and long body of mandible. To further investigate the potential linkage locus in12pter-p12.3, we performed whole-exome sequencing on the MP pedigree. No mutations were detected in the previously reported MP loci, but a mutation of *FGF23*, c.35C>A was located within the susceptibility locus 12pter-p12.3. The mutation was fully segregated with the MP phenotype, indicating that it may be the causal mutation for this pedigree.

*FGF23* contains 3 exons and encodes a protein consisting of 252 amino acids. Together with *FGF19* and *FGF21*, they belongs to a subfamily of mammalian endocrine FGFs with functions that are distinct from the other paracrine FGFs^21^,^22^. *FGF23* is most highly expressed in bone, from which it can circulate through the blood to reach its target tissues^22^,^23^. It is a key humoral regulator of vitamin D and phosphate homeostasis, which are important for bone morphogenesis^24^. For the candidate causal mutation of FGF23 p.A12D, signal peptide prediction programs indicated that it would disturb the secretory properties of FGF23. Furthermore, *in vitro* studies showed that the production of mutant FGF23 was blocked in 293T cells. Although the osteoblast maybe more suitable for *in vitro* study, the 293T cells is proper on the aim of investigating the secretory properties of a protein. Therefore, considering the known role of FGF23 in bone morphogenesis and our *in silico* and *in vitro* results, we propose that *FGF23* is likely the causal gene for the observed skeletal malformations in this MP pedigree.

Previous mouse studies show that FGF23-defective mice suffered abnormal bone development^25^,^26^. Shimada *et al* found that *FGF23* null mice had severe growth retardation with bone malformation and short life span^25^. Zhang *et al* reported abnormal skull bones (including mandible) for 1-year-old *DMP1* (Dentin matrix acidic phosphoprotein 1, regulating the FGF23 expression) null mice^26^. Although abnormal level of FGF23 could result in bone malformation, the compensation of wild-type allele of heterozygotic mice and mother-supply FGF23 through milk could reduce the harm of insufficient FGF23 during bone development^25^. The incomplete penetrance (II11 is a carrier without MP phenotype) of the studied MP pedigree might be result from the compensation of products of wild-type allele and/or mother’s milk.

The FGF23 c.35C>A mutation was also detected in 3 out of 65 cases of unrelated MP patients, which indicated that c.35C>A mutation is strongly associated with MP in China. Considering that the prevalence and linked genetic loci vary between different populations and regions, the mutation needs worldwide MP samples for further validation.

In this study, *FGF23*was identified as one of the causal genes of MP in a multiplex MP pedigree in China using linkage analysis, whole-exome sequencing, bioinformatics analyses and an *in vitro* assay. These evidence may increase the knowledge of genetic basis of MP and facilitate future investigations on etiology of this disorder.

## Materials and Methods

### Samples

A 4-generation pedigree was constructed from individuals residing in the Henan Province of China (Fig. 1A). The MP pedigree was composed of 23 living individuals and was diagnosed using lateral cephalograms in conjunction with orthodontic models. The participants were diagnosed as affected individuals if they had an ANB angle of centric jaw relationship less than 0.0°. All of them share common characteristics such as high angle of mandibular plane, long body of mandible, eversion of lower lip. None of the participants suffered from other congenital disorders. All of the 19 studied individuals provided informed consent for the biological studies. This study complies with the Declaration of Helsinki and was approved by the ethics committee of the Ethics Committee of Tongji University.

### Genotyping and genome-wide linkage scanning

We genotyped all 19 collected members using Illumina Infinium HumanLinkage-12 panel (Illumina, San Diego, CA, USA) in Beijing Institute of Genomics, Chinese Academy of Science. The panel screened 6,090 single nucleotide polymorphism (SNP) markers with an average spacing of 441-kb (0.58 cM). All reactions were performed following manufacturer’s instruction. The fluorescence signals were scanned using an Illumina BeadStation, and genotypes were assigned using the Illumina BeadStudio v3 software program.

Inconsistencies in Mendelian inheritance within the genotype data were investigated using Pedcheck. All genotype errors and markers that were found in only one genotype within the dataset were removed prior to the linkage analysis. After this initial filtering, a total of 4,958 informative autosomal SNPs were used in the linkage analysis. We performed both parametric and non-parametric linkage analyses using the software program MERLIN^27^. The parametric linkage analysis assumed an autosomal dominant model with a risk allele frequency of 0.0001, a penetrance of 0.9 for genotypes with 1 or 2 copies of the risk allele, and a phenocopy rate of 0.05.

### Whole-exome sequencing and variant calling

From the collected MP pedigree, 3 affected individuals (II5, III6, and III8) and 1 unaffected individual (II6) were chosen for whole-exome sequencing. The Agilent SureSelect Human All Exon 50 Mb kit (Agilent Technologies, Santa Clara, CA, USA) was used to capture whole exomes, and the products were resolved on an Illumina HiSeq2000 system (Illumina, San Diego, CA, USA). Paired-end sequencing with 100-bp read length was conducted on each sample. All paired reads were mapped to the human reference genome (hg19) using BWA (version 0.6-r104). PCR duplicates of the reads were removed using the Picard software program (version 1.07). The Samtools (version 0.1.18) and GATK (version 1.6) software packages were used to call variants^28^,^29^. The Pindel software program was used to detect structural variants^30^.

### Sequencing the *FGF23* in the MP pedigree and unrelated MP patients

We sequenced the *FGF23* gene in all individuals of the pedigree and in 65 unrelated MP patients (34 were female, and 31 were male, ages ranged from 14 to 58 years with an average age of 22.6). We also screened for the c.35C>A (p.A12D) mutation in 342 normal control and did not find the mutant allele in any of them.

### Predicting effects of p.A12D mutation on FGF23 function

The SignalP 4.0, PrediSi, Signal-CF, and Signal-3L tools were used to predict the effects of the p.A12D substitution on signal peptide function in FGF23. SignalP 4.0 was used to identify the signal peptide with the assumption that the protein contained no transmembrane segments. The parameters for analysis with SignalP were as follows: Organism group, Eukaryotes; D-cutoff values (optimize the performance and affect sensitivity), Default; Method, Input sequences do not include transmembrane segments. We select eukaryotic as organism group for PrediSi and Signal-CF, and Human species for Signal-3L.

### Effects of p.A12D substitution on synthesis and secretion of FGF23

#### Construction of the pcDNA3.1(+)-FGF23 expression vector

Wild-type and mutant *FGF23* DNA fragments were amplified from an *FGF23* cDNA clone (Prospec, Rehovot, Israel), digested with the Nhe I and BamH I restriction enzymes, and subcloned into the pcDNA3.1(+) vector. Sequencing confirmed that the full-length wild-type and mutant FGF23 genes had been successfully ligated into the pcDNA3.1(+) vector (Supplementary Fig. S2).

#### Transfection and qPCR

The pcDNA3.1 (+)-FGF23 vectors were transfected into human 293T kidney cell line using the Lipofectamine 2000 transfection reagent (R&S Biotechnology, Shanghai, China) according to the manufacturer’s instructions, the empty pcDNA3.1 were used as a vector control. SYBR Green-based real-time quantitative PCR (qRT-PCR) was performed using an Eppendorf Realplex real-time system with GAPDH as a reference gene. The 2^−△△CT^ method was used to calculate relative gene expression levels.

#### Immunoprecipitation and Western blotting analyses

Aliquots (500 μl) of cell platelet suspensions (4 × 10^8^ cells/ml) from transfected and untransfected 293T cell line were lysed in equivalent volumes of lysis buffer^31^. The platelet lysates (1 ml) and their corresponding culture supernatants (20 ml) were pre-cleared with protein A-agarose, immunoprecipitated with 2 μg anti-FGF23 antibody, and incubated with protein A-agarose. The immunoprecipitates (i.e., the washed protein A-agarose beads) were resolved using SDS/PAGE and electrophoretically transferred onto nitrocellulose membranes. The immunodetection of FGF23 or GAPDH was performed using mouse anti-FGF23 (1:1000) and rabbit anti-GAPDH (1:5000) primary antibodies, then added corresponding secondary antibody anti-HRP, respectively. The blots were subsequently exposed to pre-flashed photographic film.

## Additional Information

**How to cite this article**: Chen, F. *et al.* Identification of a Mutation in FGF23 Involved in Mandibular Prognathism. *Sci. Rep.* **5**, 11250; doi: 10.1038/srep11250 (2015).

## Supplementary Material

Supplementary Information

## Figures and Tables

**Table 1:** Cephalometric variables of affected members (including the carrier of II11) in studied MP pedigree.

Cephalometric measures	II3	II5	II9	II11	III2	III5	III6	III8	III9	IV1	IV2	Norms*
SNA	81.6	82.3	80.7	82.6	82.1	78.9	83.2	81.4	82.3	79.5	79.8	82.8 ± 4.0
SNB	83.2	84.6	83.1	81.7	83.6	82.3	85.6	86.7	84.9	81.6	82.1	80.1 ± 3.9
ANB	−1.6	−2.3	−2.4	0.9	−1.5	−3.4	−2.4	−5.3	−2.7	−2.1	−2.3	2.7 ± 2.0
Wits(mm)	−5.6	−7.5	−7.3	−1.5	−5.4	−6.7	−6.9	−10.3	−6.3	−7.8	−8.4	−1.2 ± 2.5
ANS-Ptm(mm)	51.6	52.9	50.9	52.1	51.9	50.2	53.2	50.7	52.8	50.5	51.1	51.1 ± 2.6
Co-Po(mm)	116.7	117.4	116.3	112.5	115.8	116.6	117.4	118.6	116.8	117.2	116.9	110.2 ± 3.8
FMA	38.9	36.8	38.2	40.6	39.8	37.6	37.7	41.5	38.5	42.6	39.5	31.1 ± 5.6

Norms*, cephalometric standards of China; ANB, anteroposterior relationship of the maxillaand mandible; SNA, anteroposterior maxillary position to anterior cranial plane; SNB, anteroposterior mandibular position to anterior cranial plane; Wits (mm), length of AO-BO distance; ANS-Ptm (mm), maxillary unit length; Co-Po (mm), mandibular unit length; FMA: the angle between the FH plane and the mandibular plane.
