# Supplementary material for: Identification of a Mutation in FGF23 Involved in Mandibular Prognathism
Source: Sci Rep. 2015 Jun 10;5:11250. doi: 10.1038/srep11250 (PMC4462018; doi:10.1038/srep11250)
Supplement: Supplementary Information [file srep11250-s1.doc]

**Identification of a Mutation in FGF23 Involved in Mandibular Prognathism**

Fengshan Chen^1,† ,*^, Qin Li^1,†^, Mingliang Gu^2^, Xin Li^3^, Jun Yu^2^, Yong-Biao Zhang^2,*^

^1^Laboratory of Oral Biomedical Science and Translational Medicine, School and Hospital of Stomatology, Tongji University, Shanghai, P. R. China.

^2^Institute of Genomics, Chinese Academy of Sciences and Key Laboratory of Genome Science and Information, Chinese Academy of Sciences, Beijing, P. R. China.

^3^Department of Cardiology, Beijing Anzhen Hospital of the Capital University of Medical Sciences, Beijing, P. R. China

Supplementary Table S1 The final left 97 variants after a series of filtering on whole-exome data.

| chr | position | sampleGenotype | FunctionDBSNP | SNP | polyPhen | granthamScore | scorePhastCons | consScoreGERP | geneList |
| --- | --- | --- | --- | --- | --- | --- | --- | --- | --- |
| 1 | 11150673 | K,G,K,K | missense | / | 0.97 | 45 | 0.992 | -2.25 | EXOSC10 |
| 1 | 12907508 | Y,T,Y,Y | missense | rs138145027 | 0 | 98 | 0.014 | -2.18 | HNRNPCL1 |
| 1 | 15987473 | Y,T,Y,Y | synonymous | rs143305830 | unknown | NA | 1 | 2.23 | RSC1A1,DDI2 |
| 1 | 19557348 | Y,C,Y,Y | missense | rs149861361 | 1 | 43 | 0.998 | 4.34 | EMC1 |
| 1 | 23779282 | R,G,R,R | intron | / | unknown | NA | 0.155 | 2.44 | ASAP3 |
| 1 | 31898756 | Y,C,Y,Y | synonymous | / | unknown | NA | 0.99 | -2.51 | SERINC2 |
| 1 | 33138361 | TTTC/T,TTTC/TTTC,TTTC/T,TTTC/T | intron | rs35897027 | unknown | unknown | 0.014 | 4.24 | RBBP4 |
| 1 | 33138364 | C/CAA,C/C,C/CAA,C/CAA | intron | rs34552890 | unknown | unknown | 0.005 | 2.68 | RBBP4 |
| 1 | 43002199 | AAG/A,AAG/AAG,AAG/A,AAG/A | frameshift | / | unknown | unknown | 0.993 | 4.59 | CCDC30 |
| 1 | 144952207 | Y,C,Y,Y | missense | rs3121544 | 0.126 | 26 | 0.993 | 0.392 | PDE4DIP |
| 1 | 151665562 | G/GT,G/G,G/GT,G/GT | intron | rs35971824 | unknown | unknown | 0.003 | 3.2 | SNX27 |
| 1 | 152280665 | R,G,R,R | missense | / | 0.001 | 74 | 0 | -1.57 | FLG |
| 1 | 183086486 | K,T,K,K | missense | rs61752348 | 0.296 | 45 | 0.992 | -1.03 | LAMC1 |
| 1 | 226342495 | M,A,M,M | missense | rs199935319 | 0 | 99 | 1 | 3.65 | ACBD3 |
| 1 | 235955172 | K,T,K,K | missense | rs199694469 | 1 | 77 | 0.998 | 5.76 | LYST |
| 2 | 24253986 | Y,C,Y,Y | missense | / | 0 | 29 | 0 | -9.65 | C2orf44 |
| 2 | 33335708 | Y,T,Y,Y | missense | rs140100437 | 0.136 | 64 | 0.993 | 5.51 | LTBP1 |
| 2 | 55544485 | M,A,M,M | synonymous | rs141894355 | unknown | NA | 0.998 | 3.17 | CCDC88A |
| 2 | 74542029 | R,A,R,R | intron | rs192966317 | unknown | NA | 0.008 | 2.58 | SLC4A5 |
| 2 | 110886686 | A/AGACCAGAT,A/A,A/AGACCAGAT,A/AGACCAGAT | intron | rs3838315 | unknown | unknown | 0.001 | 2.83 | NPHP1 |
| 2 | 112615888 | S,C,S,S | missense | rs79100806 | 0.006 | 24 | 1 | 3.2 | ANAPC1 |
| 2 | 112615933 | Y,T,Y,Y | synonymous | rs72936244 | unknown | NA | 1 | 1.18 | ANAPC1 |
| 2 | 196762491 | TA/TA,T/T,T/TA,T/TA | intron | rs5837482 | unknown | unknown | 0.997 | 5.92 | DNAH7 |
| 3 | 7340393 | Y,C,Y,Y | synonymous | rs144380165 | unknown | NA | 0.994 | -2.8 | GRM7 |
| 3 | 43389767 | K,G,K,K | missense | rs73831264 | 0.232 | 22 | 0.036 | 4.38 | SNRK |
| 3 | 44286769 | M,C,M,M | intron | / | unknown | NA | 1 | 5.05 | C3orf77 |
| 3 | 47046545 | Y,C,Y,Y | synonymous | rs187781607 | unknown | NA | 1 | 1.85 | NBEAL2 |
| 3 | 49940207 | CGT/C,CGT/CGT,CGT/C,CGT/C | frameshift | / | unknown | unknown | 1 | 4.92 | MST1R |
| 3 | 160150187 | K,T,K,K | synonymous | / | unknown | NA | 0.958 | -4.61 | SMC4 |
| 3 | 168834352 | M,A,M,M | synonymous | rs199940663 | unknown | NA | 1 | 5.88 | MECOM |
| 4 | 37863193 | Y,T,Y,Y | missense | rs76138483 | 0.19 | 155 | 1 | 4.92 | PGM2 |
| 4 | 38930985 | T/TAA,T/T,T/TAA,TAA/TAA | intron | rs35369040 | unknown | unknown | 0.002 | 3.75 | FAM114A1 |
| 4 | 41664803 | CCTTCTGTACG/C,CCTTCTGTACG/CCTTCTGTACG,CCTTCTGTACG/C,CCTTCTGTACG/C | intron | rs142890171 | unknown | unknown | 0.003 | 3.2 | LIMCH1 |
| 4 | 169835141 | Y,C,Y,Y | missense | / | 0.271 | 101 | 1 | 5.46 | PALLD |
| 5 | 106723335 | R,G,R,R | intron | rs138377566 | unknown | NA | 1 | 3.67 | EFNA5 |
| 5 | 138773222 | S,G,S,S | missense | / | 0.993 | 94 | 1 | 2.41 | DNAJC18 |
| 5 | 158511794 | G/GA,G/G,G/GA,G/GA | intron | / | unknown | unknown | 0.13 | 2.91 | EBF1 |
| 6 | 24456804 | Y,C,Y,Y | missense | / | 0.001 | 94 | 0.008 | -3.62 | GPLD1 |
| 6 | 27860621 | Y,T,Y,Y | missense | / | unknown | 29 | 1 | 4.06 | HIST1H2AM |
| 6 | 31915819 | Y,C,Y,Y | synonymous | rs117905900 | unknown | NA | 1 | 3.37 | CFB |
| 6 | 33134570 | R,G,R,R | missense | / | unknown | 98 | 0.954 | 3.89 | COL11A2 |
| 6 | 33695975 | R,G,R,R | missense | rs139720178 | 0.994 | 145 | 0.878 | 5.6 | IP6K3 |
| 6 | 43400532 | Y,C,Y,Y | missense | rs142083115 | 0.99 | 101 | 0.057 | 4.43 | ABCC10 |
| 6 | 84368843 | W,T,W,W | intron | / | unknown | NA | 0 | 2.86 | SNAP91 |
| 6 | 90577706 | TG/T,TG/TG,TG/T,TG/T | frameshift | / | unknown | unknown | 0.14 | 4.05 | CASP8AP2 |
| 6 | 90577711 | TCTTTGCCCAGACATGGA/T,TCTTTGCCCAGACATGGA/TCTTTGCCCAGACATGGA,TCTTTGCCCAGACATGGA/T,TCTTTGCCCAGACATGGA/T | frameshift | / | unknown | unknown | 0.498 | 4.33 | CASP8AP2 |
| 6 | 99860673 | Y,C,Y,Y | intron | / | unknown | NA | 0.039 | 3.75 | PNISR |
| 7 | 121651695 | K,T,K,K | synonymous | / | unknown | NA | 0.996 | 3.28 | PTPRZ1 |
| 7 | 151842355 | Y,T,Y,Y | missense | rs141106704 | 0.939 | 46 | 0.981 | 2.83 | MLL3 |
| 8 | 101721812 | R,G,R,R | missense | rs200409148 | 0.997 | 180 | 1 | 5.05 | PABPC1 |
| 8 | 101721817 | Y,T,Y,Y | missense | rs201076736 | 0.999 | 98 | 1 | 5.05 | PABPC1 |
| 8 | 101721839 | M,C,M,M | missense | rs202074479 | 0.968 | 32 | 1 | 5.05 | PABPC1 |
| 9 | 742339 | Y,C,Y,Y | synonymous | / | unknown | NA | 0.385 | -6.03 | KANK1 |
| 9 | 34725069 | W,T,W,W | missense | rs114933270 | 0 | 121 | 0.006 | 1.59 | FAM205A |
| 10 | 6143245 | S,G,S,S | synonymous | rs199867166 | unknown | NA | 0.925 | -6 | RBM17 |
| 10 | 46999189 | Y,C,Y,Y | synonymous | rs3127678 | unknown | NA | 0.002 | -5.03 | GPRIN2 |
| 10 | 46999198 | K,G,K,K | synonymous | rs3127680 | unknown | NA | 0.173 | 0.58 | GPRIN2 |
| 10 | 126631773 | K,T,K,K | synonymous | / | unknown | NA | 1 | 0.452 | ZRANB1 |
| 11 | 124189292 | Y,T,Y,Y | missense | rs74550564 | 0.139 | 21 | 0.294 | 0.921 | OR8D2 |
| 11 | 124253100 | R,G,R,R | missense | rs71491826 | 0 | 89 | 0 | -6.64 | OR8B2 |
| 12 | 4488714 | K,G,K,K | missense | / | unknown | 126 | 0.04 | -2.81 | FGF23 |
| 12 | 7172432 | R,A,R,R | synonymous | rs149258950 | unknown | NA | 0 | -8.3 | C1S |
| 12 | 8374781 | C/CACG,C/C,C/CACG,C/CACG | coding | rs71265055 | unknown | unknown | 0.003 | 0.014 | FAM90A1 |
| 12 | 58010632 | Y,T,Y,Y | synonymous | rs202098341 | unknown | NA | 0.801 | -0.754 | ARHGEF25 |
| 12 | 75816814 | G/GACA,G/G,G/GACA,G/GACA | coding | rs72459789 | unknown | unknown | 0 | 2.36 | GLIPR1L2 |
| 13 | 25021263 | Y,T,Y,Y | missense | rs77269056 | 0.997 | 43 | 0.999 | 4.71 | PARP4 |
| 13 | 28626718 | W,A,W,W | missense | / | 0.075 | 149 | 0 | -7.52 | FLT3 |
| 14 | 23548783 | A/AGAACGT,A/A,A/AGAACGT,A/AGAACGT | coding | rs34293824 | unknown | unknown | 1 | 5.68 | ACIN1 |
| 14 | 76368661 | R,G,R,R | intron | rs150594372 | unknown | NA | 0.011 | 4.54 | TTLL5 |
| 14 | 77935439 | S,C,S,S | synonymous | / | unknown | NA | 0.997 | -0.322 | AHSA1 |
| 14 | 102483555 | K,T,K,K | missense | rs201272954 | 1 | 109 | 0.83 | 5.46 | DYNC1H1 |
| 15 | 28447562 | R,A,R,R | missense | rs146883683 | 0 | 74 | 0.801 | 3.07 | HERC2 |
| 15 | 42714195 | G/GA,G/G,G/GA,G/GA | intron | / | unknown | unknown | 0.047 | 2.72 | ZFP106 |
| 15 | 52613630 | M,A,M,M | missense | rs199788926 | 1 | 109 | 1 | 4.68 | MYO5A |
| 15 | 71276480 | GCAA/G,GCAA/GCAA,GCAA/G,GCAA/G | coding | rs3834543 | unknown | unknown | 1 | 4.17 | LRRC49 |
| 16 | 3808052 | TA/T,TA/TA,TA/T,TA/T | intron | rs75459669 | unknown | unknown | 0.885 | -0.298 | CREBBP |
| 16 | 30006689 | M,A,M,M | missense | rs200916858 | 0.995 | 109 | 0.993 | 4.67 | HIRIP3 |
| 16 | 30999245 | K,T,K,K | missense | rs201481942 | 0.001 | 109 | 0.998 | 5.1 | HSD3B7 |
| 16 | 70182375 | Y,T,Y,Y | synonymous | rs62050977 | unknown | NA | 0.987 | -0.306 | PDPR |
| 16 | 70182390 | K,T,K,K | missense | rs62050978 | 0.037 | 94 | 0.763 | -1.15 | PDPR |
| 17 | 4109643 | Y,T,Y,Y | missense | rs200196247 | 0.996 | 56 | 1 | 5.5 | ANKFY1 |
| 17 | 21319171 | R,G,R,R | missense | rs73313922 | 1 | 23 | 1 | 5.32 | KCNJ12 |
| 19 | 7987522 | Y,C,Y,Y | missense | / | 0.503 | 98 | 0.011 | 0.598 | SNAPC2 |
| 19 | 8601222 | Y,C,Y,Y | missense | rs199663368 | 0.19 | 29 | 0.873 | 3.44 | MYO1F |
| 19 | 9076053 | AC/A,AC/AC,AC/A,AC/A | frameshift | / | unknown | unknown | 0.011 | 0.881 | MUC16 |
| 19 | 31769725 | S,G,S,S | missense | rs199546310 | 0.464 | 112 | 0.069 | 4.43 | TSHZ3 |
| 19 | 34302354 | Y,C,Y,Y | missense | rs144406308 | 0.002 | 89 | 0.996 | 5.01 | KCTD15 |
| 19 | 38385079 | Y,C,Y,Y | missense | rs150405572 | 0.983 | 56 | 0.909 | 2.54 | WDR87 |
| 19 | 38851193 | K,T,K,K | missense | / | 0.718 | 109 | 0 | 2.48 | CATSPERG |
| 19 | 40363913 | R,A,R,R | missense | / | 0.999 | 98 | 0.382 | 5.34 | FCGBP |
| 19 | 40376675 | R,G,R,R | missense | rs77872255 | 0.898 | 64 | 0.897 | -0.612 | FCGBP |
| 19 | 40419842 | R,A,R,R | missense | / | 0.047 | 98 | 0.7 | 3.74 | FCGBP |
| 19 | 49248538 | R,G,R,R | synonymous | / | unknown | NA | 0.001 | -0.076 | IZUMO1 |
| 19 | 51920196 | K,G,K,K | missense | rs73049612 | 0.765 | 53 | 0.32 | 2.57 | SIGLEC10,LOC100129083 |
| 20 | 4680521 | R,G,R,R | missense | rs1800014 | 0.562 | 56 | 0.761 | 3.14 | PRNP |
| 20 | 43535145 | TA/TA,T/T,TA/TA,T/TA | utr-3 | rs11468357 | unknown | unknown | 0.991 | 2.99 | YWHAB |
| 22 | 46654664 | K,G,K,K | missense | rs148258530 | 0.574 | 77 | 0 | -8.57 | PKDREJ |

SNP, rsID of the variant; Chr, Chromosome; Position, physical position according to hg19 reference genome; FunctionDBSNP, dbSNP class of variation function; polyPhen, amino acid substitution impacts; granthamScore, the Grantham score of any amino acid changes; scorePhastCons, conservation Score (range of 0 to 1, with 1 being the most conserved) phastCons predicted from 46 placental mammalian species; consScoreGERP, conservation Score GERP is a rejected-substitution score (range of -12.3 to 6.17, with 6.17 being the most conserved) from the program GERP. geneList, the gene that the SNP located.

| Supplementary Table S2 Identified SNPs in the *FGF23* gene with the MP pedigree and 65 unrelated MP patients. | | | | | | | | |
| --- | --- | --- | --- | --- | --- | --- | --- | --- |
|  | SNP1 | SNP2 | SNP3 | SNP4 | SNP5 | SNP6 | SNP7 | SNP8 |
| rsID | rs71534281 | rs13312794 | rs7955866 | / | rs11063118 | rs3832879 | / | rs3812822 |
| Allele(Major/Minor) | C/G | T/G | C/T | A/C | A/G | InsC | C/A | T/C |
| Position (hg19) | 4479480 | 4479486 | 4479549 | 4479754 | 4481564 | 4481898 | 4488714 | 4489235 |
| gene positon | exon3 | exon3 | exon3 | exon3 | intron2 | intron1 | exon1 | 5'near gene |
| Functional region | 3'UTR | 3'UTR | missense | missense | / | / | missense | / |
| mRNA position | / | / | c.716C>T | c.511A>C | / | / | c.35C>A | / |
| coden | / | / | ACG-ATG | ACC-CCC | / | / | GCC-GAC | / |
| polypeptide | / | / | Thr239-to-Met | Thr171-to-Pro | / | / | Ala12-to-Asp | / |
| genotype of related MP patients | 64/1/0 | 60/5/0 | 44/19/2 | 65/0/0 | 44/18/3 | 61/4/0 | 65/0/0 | 42/20/3 |
| genotype of the MP pedigree | 19/0/0 | 15/4/0 | 8/1/0 | 18/1/0 | 15/4/0 | 19/0/0 | 9/10/0 | 18/1/0 |

Supplementary Table S3 The minor allele frequency of SNPs in *FGF23* in genotyped populations from 1000 genomes and NHLBI GO Exome Sequencing Projects (ESP).

| project | population | SNPs | | | | | | | |
| --- | --- | --- | --- | --- | --- | --- | --- | --- | --- |
|  |  | rs71534281 | rs13312794 | rs7955866 | FGF23 c.511A>C | rs11063118 | rs3832879 | FGF23 c.35C>A | rs3812822 |
| 1000 Genomes Project | ASW | 0 | 0 | 0.1066 | / | 0.1557 | 0.0082 | / | 0.1557 |
|  | CEU | 0 | 0 | 0.1235 | / | 0.2294 | 0 | / | 0.1176 |
|  | CHB | 0 | 0.0412 | 0.1959 | / | 0.2268 | 0 | / | 0.2010 |
|  | CHS | 0 | 0.0400 | 0.205 | / | 0.2450 | 0 | / | 0.2200 |
|  | CLM | 0 | 0.0250 | 0.1917 | / | 0.2917 | 0 | / | 0.1917 |
|  | FIN | 0 | 0 | 0.1129 | / | 0.3226 | 0 | / | 0.1183 |
|  | GBR | 0 | 0 | 0.1292 | / | 0.2528 | 0 | / | 0.1292 |
|  | IBS | 0 | 0 | 0.2143 | / | 0.3929 | 0 | / | 0.2143 |
|  | JPT | 0 | 0.0449 | 0.0955 | / | 0.1629 | 0 | / | 0.0955 |
|  | LWK | 0 | 0.0104 | 0.1198 | / | 0.1823 | 0 | / | 0.1823 |
|  | MXL | 0 | 0.0547 | 0.1328 | / | 0.2422 | 0 | / | 0.1406 |
|  | PUR | 0 | 0 | 0.2091 | / | 0.2727 | 0 | / | 0.2091 |
|  | TSI | 0.0051 | 0.0051 | 0.0969 | / | 0.2296 | 0 | / | 0.0816 |
|  | YRI | 0 | 0.0227 | 0.0909 | / | 0.1136 | 0 | / | 0.1250 |
| ESP | EA | 0.0017 | 0.0003 | 0.1131 | / | / | 0.1236 | / | / |
|  | AA | 0 | 0.0161 | 0.0901 | / | / | 0.0281 | / | / |

"/" represents the SNP was not reported in corresponding population. ESP, NHLBI GO Exome Sequencing Project; ASW, Americans of African Ancestry in SW USA; CEU, Utah Residents (CEPH) with Northern and Western European ancestry; CHB, Han Chinese in Bejing, China; CHS, Southern Han Chinese; CLM, Colombians from Medellin, Colombia; FIN, Finnish in Finland; GBR, British in England and Scotland; IBS, Iberian population in Spain; JPT, Japanese in Tokyo, Japan; LWK, Luhya in Webuye, Kenya; MXL, Mexican Ancestry from Los Angeles USA; PUR, Puerto Ricans from Puerto Rico; TSI, Toscani in Italia; YRI, Yoruba in Ibadan, Nigera; EA, European American; AA, African American


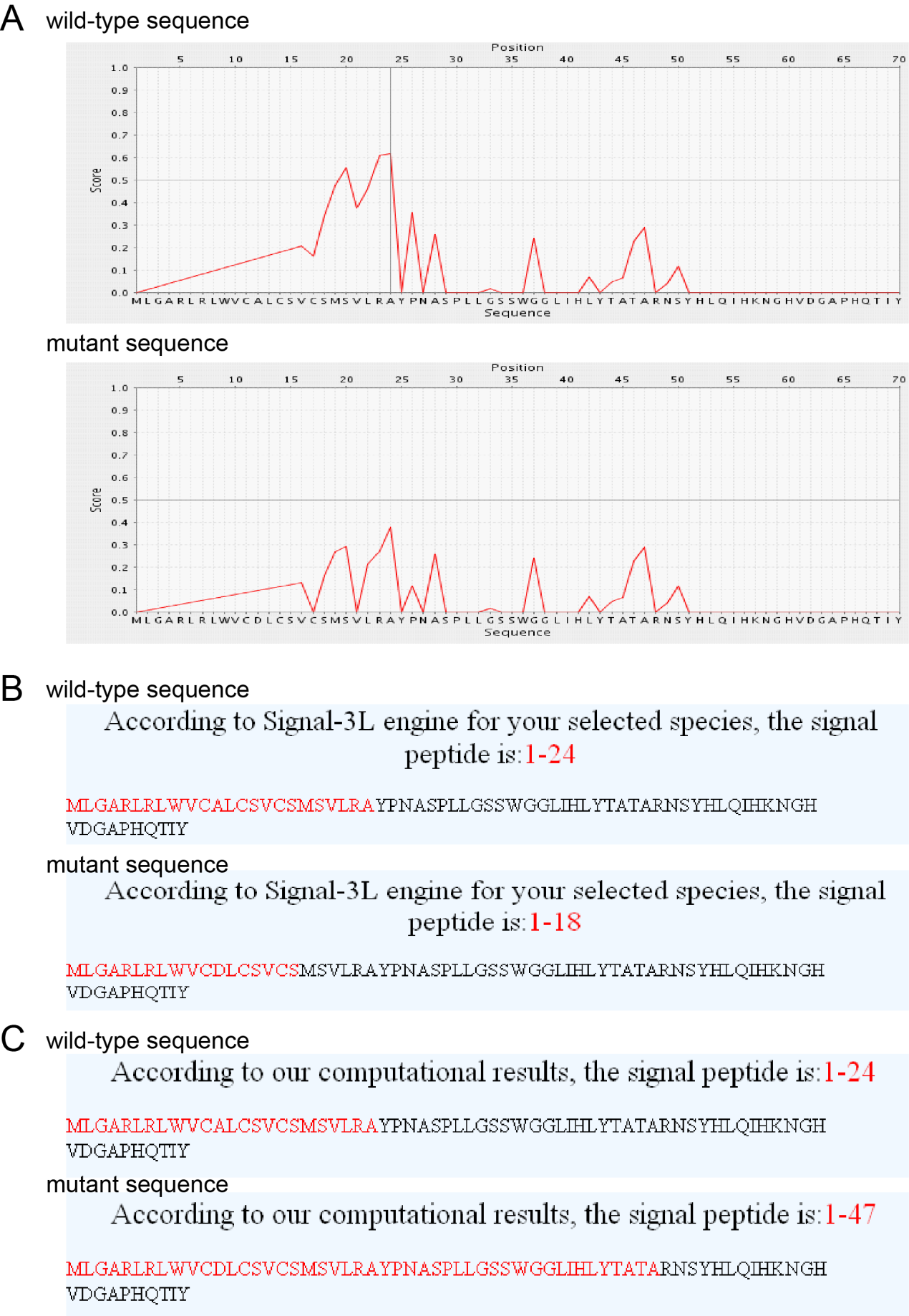


**Supplementary Fig. S1 Signal peptide prediction in the wild-type and mutant FGF23 proteins.** PrediSi predicted that the mutant FGF23 protein would not be secreted. Signal-3L and Signal-CF predicted that it would have 2 signal-peptide cleavage sites at amino acids 19M and 48R, whereas wild-type FGF23 would have a single cleavage site at 25Y. Panels (A), (B), and (C) show the predicted signal peptides from PrediSi, Signal-3L, and Signal-CF, respectively.


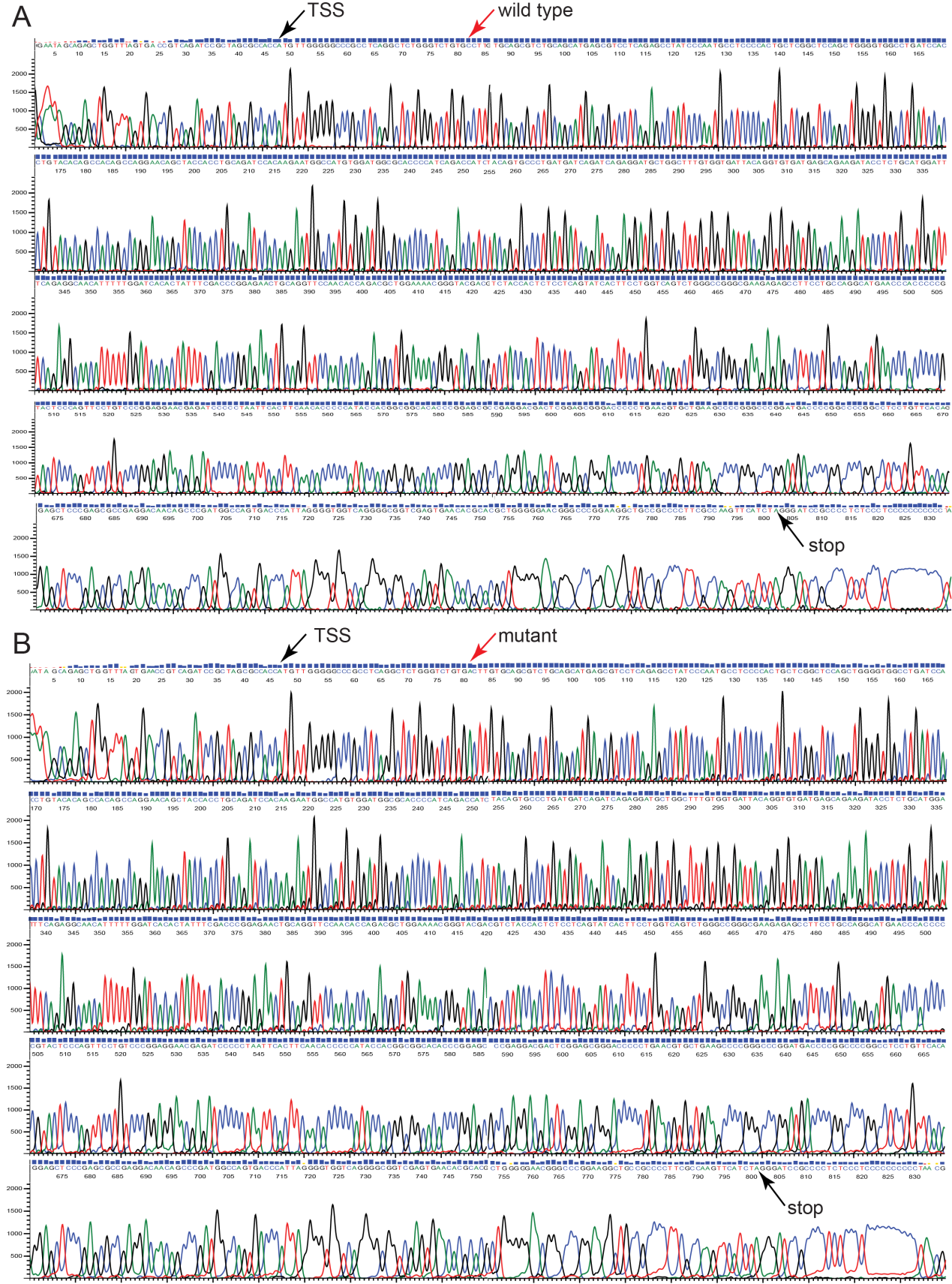


**Supplementary Fig. S2 The wild-type and mutant *FGF23* sequences were inserted into the pcDNA3.1(+) vector.** We sequenced the recombinant constructs to confirm the integrity of the wild-type and mutant *FGF23* inserts. (A) Wild-type *FGF23*. (B) Mutant *FGF23*.
